# Postnatal Repair of Open Neural Tube Defects: A Single Center with 90-Month Interdisciplinary Follow-Up

**DOI:** 10.3390/jcm10194510

**Published:** 2021-09-29

**Authors:** Christian Hagemann, Kara Krajewski, Thomas Henne, Ralf Stücker, Philip Kunkel

**Affiliations:** 1 Department of Pediatric Neurosurgery, Altona Children’s Hospital, Bleickenallee 38, 22763 Hamburg, Germany; Christian.Hagemann@kinderkrankenhaus.net (C.H.); Philip.Kunkel@umm.de (P.K.); 2 Department of Pediatric Nephrology, Altona Children’s Hospital, Bleickenallee 38, 22763 Hamburg, Germany; Thomas.Henne@kinderkrankenhaus.net; 3 Department of Pediatric Orthopedics, Altona Children’s Hospital, Bleickenallee 38, 22763 Hamburg, Germany; Ralf.Stuecker@kinderkrankenhaus.net; 4 Department of Pediatric Neurosurgery, University Medical Center Mannheim, 68135 Mannheim, Germany

**Keywords:** postnatal repair of myelomeningocele, open neural tube defects, fetoscopic surgery, prenatal surgery

## Abstract

After publication of the Management of Myelomeningocele Study (MOMS) there is confusion regarding which treatment of open neural tube defects (NTD) is best. We report our results of postnatally repaired open NTDs born between 2007–2018 (*n* = 36) in critical reflection of the MOMS study. Neurosurgical, orthopedic, and urologic data were assessed. We also introduce a new entity: “status post prenatal repair”. FU ranged from 29 to 161 months (mean: 89.1 m) in 7 cases of myeloschisis and 24 myelomeningoceles in the final collective *n* = 31. The shunt rate was 41.9%, and the endoscopic third ventriculostomy rate was 16.1%. Hydrocephalus requiring treatment was not associated with the anatomical level, but with premature birth (*p* = 0.048). Myeloschisis was associated with shunt placement (*p* = 0.008). ROC analysis revealed birth <38.5th week predicts the necessity for hydrocephalus treatment (sensitivity: 89%; specificity: 77%; AUC= 0.71; *p* = 0.055). Eight (25.8%), patients are wheelchair-bound, 2 (6.5%) ambulate with a posterior walker, 10 (32.3%) with orthosis and 11 (35.5%) independently. One (3.2%) patient underwent detethering at 5.5 years. A total of three patients underwent five Chiari decompressions (9.6%). Further, nineteen orthopedic procedures were performed in nine patients (29.0%). A total of 17 (54.8%) patients self-catheterize, which was associated with an anatomical lesion at L3 or below (*p* = 0.032) and 23 (74.2%) take anticholinergic medication. In conclusion, shunt dependency is associated with myeloschisis, not with the anatomical defect level. Hydrocephalus treatment is associated with premature birth. In this postnatal cohort with significantly longer follow-up data than the MOMs study, the ambulation rate is better, the shunt rate lower and the secondary tethered cord rate better compared to the MOMS study.

## 1. Introduction

Myelomeningocele and myeloschisis are common open neural tube defects (NTD) and are associated with significant morbidity related to hydrocephalus, incontinence, inhibited ambulation, and lifelong subsequent neurosurgical, orthopedic, and nephro-urologic management. The “double hit hypothesis” was established by Heffez DS et al. and proposed the impact to occur in two steps: first, the neural tube defect itself occurs during neurulation, and secondly, the continuous contact of the placode with amniotic fluid causes further damage [1].

The Management of Myelomeningocele Study (MOMS), first published in 2011, has brought answers and questions regarding optimal treatment for open NTD [2]. To avoid secondary damage to the placode as described above, the strategy of prenatal closure was transferred to humans after being investigated in animal models. The study was stopped due to their significant findings in favor of prenatal treatment after enrolling 183 patients with respect to shunt rate, hindbrain herniation, secondary tethered cord and independent ambulation [2].

Nevertheless, Meuli and Moehrlen published their opinion that MOMS “has indisputedly shown that overall, open prenatal repair is distinctly better than postnatal care alone” and “prenatal repair is the novel standard of care” [3]. We question this conclusion because of the limited data published in MOMS as our experience with postnatal repair differs greatly. Further, a review by the Cochrane database revealed the conclusions in favor of prenatal repair to be insufficient [4]. Further, a recent postnatal closure cohort demonstrated superior results to MOMs with respect to ambulation, anatomic-functional level on longer follow-up [5].

We consider it necessary for prenatal maternofetal surgery to be better or at least equal than postnatal repair, as otherwise risking mother and fetus is unacceptable [6].

We therefore performed a retrospective analysis of 36 open NTDs treated primarily at our institution from 2007–2018 and compare the results with the MOMS trial as well as a recent series of patients treated fetoscopically. Further, we introduce a new diagnosis we have termed “status post prenatal repair” and report on our eight patients with this entity.

## 2. Materials and Methods

All consecutive patients undergoing postnatal open NTD repair between 2007 and 2018 at our institution were identified retrospectively through the hospital coding system. Birth-related, clinical, neurosurgical, orthopedic and nephrourological data were obtained from the electronic patient records, which included outpatient visit summaries and radiological data. Gestational age of, i.e., 38 weeks was defined as 38 0/7. Patients with status post prenatal repair were identified after reviewing our outpatient clinic lists, as “status post prenatal repair” does not have its own diagnosis code to date. The electronic patient record was similarly utilized for all relevant information. Data were anonymized for further analyses. Ethics approval by the local ethics committee was obtained (Ethik-Kommission der Ärztekammer Hamburg, 2021-300033-WF). Informed consent was not required according to local ethics committee regulations (Hamburger Krankenhausgesetz §12).

### 2.1. Presurgical Treatment

**Figure 1:**
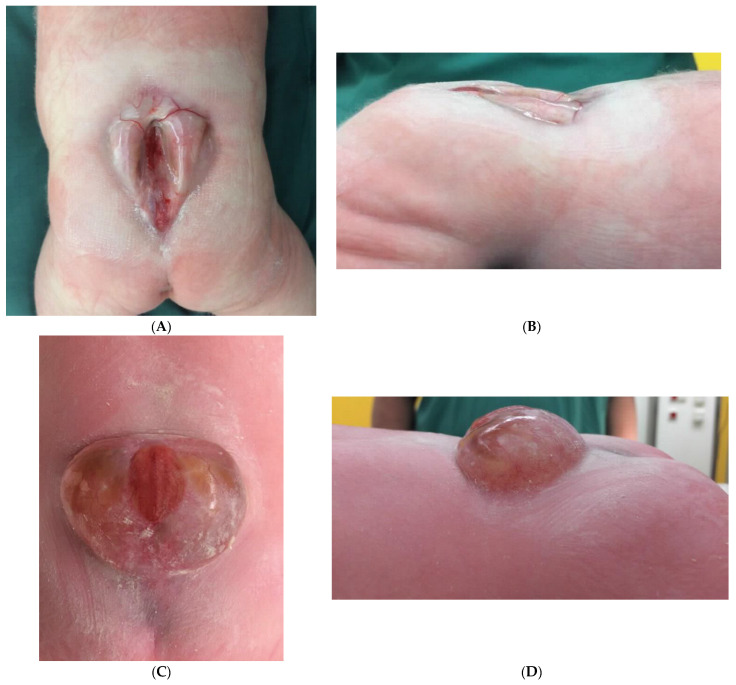
Typical appearance of a myeloschisis (**A**,**B**) and myelomeningocele (**C**,**D**). Myelomeningoceles are easier to detect prenatally due to the presence of a dorsal sack.

All patients were evaluated clinically with respect to their functional level and categorized as myelomeningocele or myeloschisis, see Figure 1. The NTD was covered with a sterile bandage and patients were placed strictly in prone position before surgical repair.

### 2.2. Surgical Repair

We consider a microsurgical, multilayer repair after dissection of the placode and neurulation of utmost importance in order to avoid secondary tethered cord as well as epidermoid formation. A filum terminal should be transected. Neurulation is performed with 8-0 interrupted sutures; dura repair is performed with 6-0 continuous sutures. All defects were repaired directly or with pedicled skin flaps; free skin flaps were never used.

### 2.3. Postoperative Care

**Figure 2:**
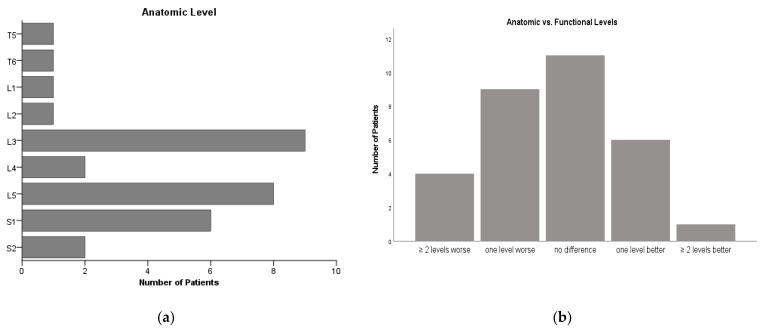
Frequency distribution of the anatomic defect levels (**a**) in our cohort as well as anatomical vs. functional levels (**b**).

The functional level of the defect was documented postoperatively. In some cases, there are different functional levels in each limb; see the Results section and Figure 2. All patients were followed by a multidisciplinary team of pediatric neurosurgeons, orthopedic surgeons, nephrologists, pediatricians, physiotherapists, and orthopedic technicians. Table 1 summarizes the treatment algorithm for our MMC patients and Table 2 summarizes the indications for further surgeries.

### 2.4. Statistics

The data were collected and anonymized before statistical evaluation. The statistical analyses were performed using SPSS (IBM SPSS Statistics, Version 27, Chicago, USA). Chi-square (Fisher’s exact) and ROC analyses were used to determine the association between risk factors and hydrocephalus treatment. A *p*-value ≤ 0.05 was regarded as statistically significant; however, all statistical tests are to be considered as non-confirmative.

## 3. Results

A total of 36 patients underwent postnatal open neural tube defect repair: 29 (80.6%) myelomeningeoceles and 7 (19.4%) myeloschisis. A total of 19 patients were male and 17 were female. NTD was detected prenatally in 20/31 (64.5%) of patients. Further, 25/31 (80.6%) patients were delivered via cesarean section. Average gestation was 37 + 4 weeks. Two patients died due to extreme prematurity (26th week, 810 g, and 24th week, 740 g). Three patients were lost to follow-up. Therefore, 31 patients were available for analysis. Follow-up ranged from 29 to 161 months (mean: 89.1 months).

In four cases, secondary wound healing after primary repair was necessary, but no additional surgical procedures, i.e., free flap, were required. In one case, revision was performed due to cerebrospinal fluid fistula on Day 4. In 3/31 (9.7%) patients, overall six additional NTDs were detected: two split cord malformations, one limited dorsal myeloschisis, one encephalocele, and two intrathecal lipomas. Half of the additional NTDs were addressed in the initial session, half were treated later.

Figure 2 demonstrates the anatomical defect levels as well as the anatomical vs. functional levels. Nine patients had different functional levels in each limb (eight varied by one level, one by two levels).

### 3.1. Hydrocephalus

A total 18/31 (58.1%) patients developed hydrocephalus that met the criteria for surgical treatment; 13 underwent ventriculoperitoneal shunting within the first three months of life (41.9%) and 5 (16.1%) patients underwent ETV after their first birthday. No patients developed shunt criteria in between. Overall, 13 patients with postnatal open NTD repair did not meet the criteria for surgical treatment to date (41.9%). Hydrocephalus requiring treatment was not associated with anatomical level. However, shunt placement was strongly associated with myeloschisis (*p* = 0.008). Further, 6/7 (85.7%) myeloschisis patients underwent shunt placement, whereas 7/24 (29.2%) myelomeningoceles underwent shunt placement. All ETVs were performed in myelomeningoceles, and none in myeloschisis.

Receiver operating curve analysis revealed birth prior to the 38 + 4th week predicts the necessity for hydrocephalus treatment with a sensitivity of 89% and a specificity of 77% (AUC= 0.71; *p* = 0.055, asymptotic). Basic Chi-square analyses similarly revealed a direct correlation between premature birth (before the 38th week) and hydrocephalus treatment (*p* = 0.053, exact).

Birth by cesarean section and lack of prenatal detection were not associated with hydrocephalus treatment, respectively. Anatomical level above/below L3 was also not associated with shunt placement or hydrocephalus treatment.

Further, ten of the thirteen patients with a shunt developed complications that resulted in shunt revision (76.9% of the shunt patients, in eight cases due to mechanical issues, in two cases due to shunt infection). The shunt revisions were performed 3–86 months after shunt implantation.

### 3.2. Chiari II

A total of 30/31 patients manifested Chiari malformation on MRI, including one case of additional encephalocele and three cases of cerebellar hypoplasia. In three patients, the fourth ventricle was below the foramen magnum. A total of five Chiari decompressions were performed in three children (9.7% of patients). One case with severe caudal cranial nerve palsies underwent decompression at 2, 3, and 4 months of age due to swallowing difficulties and pneumonia. She underwent transient tracheostomy, which could be removed at age 18-months, and she was able to eat independently. In one patient, the hindbrain decompression was indicated at 34 months of age. In this particular case, central apnea in sleep studies lead to surgery.

### 3.3. Secondary Tethered Cord

Although all children presented signs of tethered cord on MRI studies, only one patient developed symptomatic secondary tethered cord, which led to detethering surgery at age 5.5 years (3.2%). In this case (L1 defect level with L2/L4 split functional levels), slowly progressive spasticity of lower limbs and vesicouretic reflux with bladder trabeculation were the clinical signs of tethered cord.

### 3.4. Additional NTDs

Six additional NTDs were detected in three patients (9.7%): two split cord malformations, one limited dorsal myeloschisis, one encephalocele, and two intrathecal lipomas. Half of the additional NTDs were addressed during the initial session, half were treated later.

### 3.5. Nephro-Urological Follow-Up

Seven urologic procedures were performed in five patients: two temporary suprapubic catheters, one urethra lengthening, one appendicostomies (Malone procedure), and one ureterocystoneostomy (Politano-Leadbetter procedure). Urologic procedures were performed at age 0.5–80 months. Currently, no urinary diversion had been necessary.

A total of 17/31 patients (54.8%) perform self-catheterization, which was significantly associated with an anatomical lesion at L3 or below (*p* = 0.032). Further, 23/31 (74.2%) currently take anticholinergic medication.

### 3.6. Orthopedic Follow-Up

**Figure 3:**
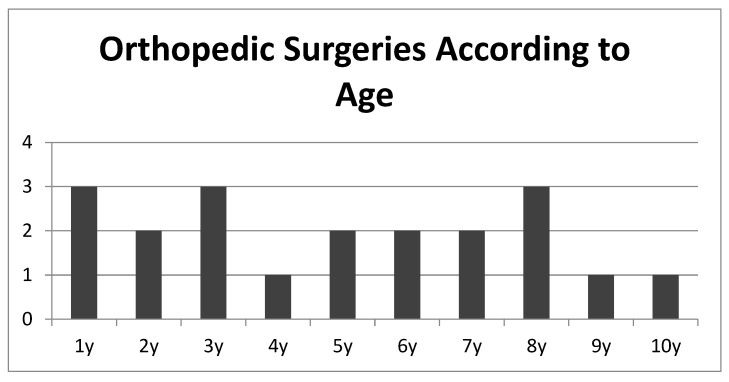
Orthopedic procedures according to patient age. Orthopedic procedures have to be expected in all years of growth in open NTDs corresponding to Marreiros et al. [5]. Therefore, orthopedic FU till adulthood is necessary.

A total nineteen orthopedic procedures were performed in nine cases (29.0%). Four patients lacked orthopedic follow-up data as they are undergoing orthopedic follow-up at other institutions. Among those treated at our hospital, the following procedures were performed on the lower limbs: seven hip procedures, one Evans procedure, two tenotomies, one epiphyseodesis, one surgery for peri-implant fracture, one derotational osteotomy, and two removals of osteosynthesis material. With respect to spinal procedures, two hemivertebra resections and two vertical expendable prosthetic titanium ribs (VEPTR) implantations were performed. VEPTR were implanted due thoracic insufficiency syndrome associated with scoliosis and these two cases subsequently underwent nine VEPTR distractions. One 11-year-old patient was recently diagnosed with progressive scoliosis and is scheduled to undergo detethering followed by VEPTR implantation. In some cases, orthopedic findings were not yet addressed, and further FU will make additional procedures necessary. Orthopedic procedures were performed between the ages of 2–128 months (Figure 3).

### 3.7. Ambulation

Eight patients are wheelchair bound (25.8%), two ambulate with a posterior walker (6.5%), ten (32.3%) with orthosis and eleven (35.5%) are able to ambulate independently. All independent walkers (*n* = 11) had lesions at or below L3; ambulation ability in general was significantly associated with anatomic level (*p* = 0.02).

### 3.8. Status Post Prenatal Repair

**Figure 4:**
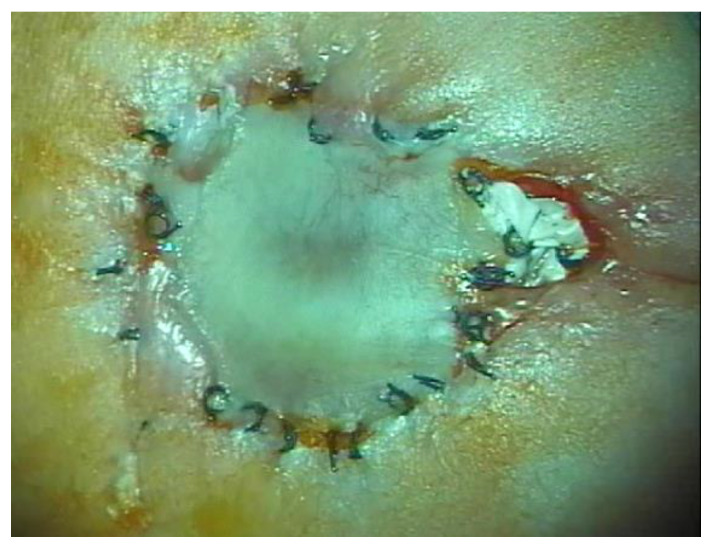
Patient No. 3 in the status post prenatal repair group at birth; immediate surgery was indicated due to a CSF fistula.

We have treated eight patients with status post prenatal repair from various institutions in Germany and Switzerland (see Table 3). Patient No. 2 underwent fetosurgical repair, the method of repair is unclear for patient No. 6, and the remainder underwent patch placement fetoscopically. Treatment of complications are not offered at many of the centers for prenatal MMC treatment. We have followed these patients an average of 76 months. Five of the eight (62.5%) have undergone treatment for hydrocephalus. All have radiological evidence of Chiari and are being closely monitored; however, no patients have undergone decompression for Chiari to date. Only one patient (12.5%) can ambulate freely. Further, one patient is wheelchair-bound and the remainder required orthosis and/or a walker (75.0%). Figure 4 demonstrates a typical patch placement with CSF fistula.

## 4. Discussion

### 4.1. Morbidity and Mortality

The disadvantages of prenatal repair seem to be prematurity and the necessity of fast closure of the defect to avoid fetomaternal risks. Fetal deaths have been reported after prenatal repair, both fetoscopically and fetosurgically [7,8,9,10]. The MOMS follow-up paper reported a 5.5% death rate in the prenatal repair vs. 3.2% in the postnatal cohort [11]. Diehl et al. recently reported a death rate of 5.6% in their fetoscopic series [12]. The two cases of fatality in the present cohort were due to extreme prematurity for other reasons. Average gestational age for MOMS children was 34GW (prenatal) vs. 37GW (postnatal) [2], which was similar to our postnatal cohort (37 + 4). The recent fetoscopic cohort reported a mean gestational age of 33 + 1 weeks, with 11.5% born prior to the 30th week [12]. Unfortunately, very little data are provided on the latter cohort regarding prematurity-related morbidity.

Further, subsequent pregnancies after hysterotomy are associated with a higher risk of uterine rupture, which might be of lesser concern in fetoscopic patch repair [13]. A systematic review on fetal and maternal risks in prenatal treatment came to this conclusion: serious maternal complications occur in 1.7% fetoscopic and 4.5% open hysterotomy procedures [14]. Prematurity after maternofetal surgery is a major concern, in particular as a 5% rate of periventricular leukomalacia has been reported [2].

Maternal risks are significant for prenatal surgery with complications occurring in 20.9% open fetal surgeries including serious complications in 4.5%; therefore, many ethical questions are arising [14,15].

### 4.2. Hydrocephalus

In historic reports published until 1990, many preferred shunt implantations at birth, even when head circumference was within normal limits [16]. Thus, a higher shunt rate is to be expected in historic cohorts. Januschek et al. reported an 85% shunt rate and Laskay et al. an 84.6% shunt rate in postnatal repair [17,18]. The National Patient Spina Bifida Registry (79% shunt rate) published a similar shunt rate in their publications as the postnatal cohort of MOMS [2,19]. Marreiros et al. reported 70% in their cohort of more than 80 cases [20]. Our parameters for shunt implantation were the same as MOMS, with the exception of applying ETV in patients over 12 months. Nevertheless, our shunt rate is only 41.9%, which is lower than reported by Hilton et al. in their FU for prenatal repair [21]. Further, it is even lower than the prenatally treated children in the MOMs series (49%) [11] and the recent fetoscopic series (48.1%) [12]. The fetoscopic collective even included strict prenatal criteria which excluded large ventricles on prenatal ultrasound [12]. We are not the only center with lower shunt rates in postnatal repair: Chakraborty et al. reported 51.9% for 54 cases in 2008, although they did not perform ETV [22]. It is of great benefit to be shunt independent as the revision rate in NTD is particularly high, in our cohort nearly 80% on our long FU. Therefore, in affected patients over 12 months, we recommend ETV. To indicate ETV in affected infants older than 12 months is a step forward, and to date, after a mean of 87 months FU (range: 56–128) on *n* = 5 ETV patients, none have required conversion to a shunt. Neither MOMs nor the most recent fetoscopic collective report on ETV.

We found hydrocephalus treatment overall not to be dependent on anatomical level, but on myeloschisis, which supports the theory for development of Chiari due to persistent loss of CSF during pregnancy. Unfortunately, the MOMS does not distinguish between myeloschisis and myelomeningocele. Further, we are the first to show a significant association between gestational week and hydrocephalus treatment.

### 4.3. Chiari II

If the loss of CSF results in Chiari malformation, and MOMS reported less hindbrain herniation after prenatal repair, we would expect the Chiari decompression rate in the postnatal group to be higher than 11% as reported by MOMS [11]. Marreiros et al. reported 9.5% Chiari decompression in their series with FU longer than 12 months [20]. Unfortunately, Chiari decompression rate after fetoscopic prenatal repair is not reported. In our cohort, 9.7% underwent Chiari decompression, but only two cases within the first year of life, therefore longer FU is needed for meaningful comparisons. Further, we define criteria to indicate Chiari surgery based on several factors, including sleep studies, whereas many others, including MOMs and Diehl et al. only define radiological hindbrain herniation without clinical criteria. MOMs did report 4% Chiari decompression in their prenatal group [11]; however, it is unclear what the indication for surgery is based upon, as explained above.

### 4.4. Secondary Tethered Cord

It is unlikely that in utero surgery of open NTD enables reconstruction of the anatomic layers as performed in postnatal repair, where neurulation is performed and a filum is transected, followed by closure of all the layers. This is reflected in the MOMS follow-up paper with a 27% rate of detethering in the prenatal group vs. 15% in the postnatal group [11]. Most of postnatal repairs at our institution take 2 to 4 h depending on the size of the defect and availability of skin and soft tissue. Such a repair may offer the chance to avoid secondary tethered cord, syringomyelia, or epidermoid formation in the future. Accordingly, our detethering rate was 3.2%. Simply placing a patch directly on a placode via fetoscopy does not seem reasonable for most neurosurgeons and we consider the rate of secondary tethered cord as high, but there are no reports regarding this matter despite the use of this method since 2008 [12,23,24].

Adzick et al. reported 8% detethering in prenatal versus 1% in postnatal group, but only up to 12 months [2]. Marreiros et al. reported 45% detethering surgeries in their longtime FU [20]. We have already performed detethering in 25.0% of our status post prenatal group, despite shorter follow-up than our postnatal series.

### 4.5. Nephrourological

Nephrourological FU is lacking in the most recent fetoscopic series [12]. The rate of clean intermittent catheterization (CIC) at 6 years is 62% vs. 87% in the prenatal and postnatal groups for MOMs, respectively, as reported by Brock JW et al. in 2019 [25]. In comparison, our cohort has a CIC rate of only 54.8%, which is lower than the prenatal cohort of the MOMs study. This can be an indication of better urological outcome or may be due to varying continence management strategies among families. Further, we were able to show a significant association between anatomical lesion at or below L3 and CIC. Our cohort had 87% lesions at or below L3, which is comparable to 82% in both the pre- and postnatal cohorts in MOMs [11].

### 4.6. Orthopedic

Marreiros et al. reported 20% pathologic kyphosis in their longtime FU [20]. Comprehensive orthopedic FU data are lacking for many other studies including MOMs as well as the recent fetoscopic cohort [12]. We work very closely with our orthopedic colleagues as orthopedic FU is essential for well-being and quality of life as well as ambulation and even ventilation, with respect to scoliosis.

### 4.7. Independent Ambulation

In our cohort, 35.5% walk independently, compared to 29% in the prenatal MOMs cohort on school-age follow-up [11] and 46% in the recent fetoscopic collective [12], though their prenatal selection criteria were quite narrow.

### 4.8. Status Post Prenatal Repair

As neurosurgeons, we are now, in fact, faced with a new entity to treat. There are two prenatal methods implemented: fetoscopic patch placement and fetosurgical repair. They present similar problems for postnatal care, which is exclusively in pediatric neurosurgical hands for 18 years, which prompted the authors to propose a new official diagnosis of this iatrogenic disease.

When patients are fetoscopically treated with a patch over the defect, it is not only extremely difficult to repair cases of CSF leakage in a newborn with a patch (which is extremely unphysiological material that completely masks the normal anatomy, so that we cannot adapt the appropriate layers and perform true neurulation with watertight duroplasty), but children who have been patched and develop tethered cord later in life are at a much higher risk of neurological deficits after surgery because the anatomy was not readapted but is stuck together with scar tissue as the result of a patch being placed on the defect. A filum terminal is not trans-sected in this method, so that detethering later in life is highly likely.

Even the children treated fetosurgically have many problems because the prenatal closure must be performed within an hour, approximately, in order to avoid obstetric complications. A very hasty closure (instead of a meticulous postnatal closure which can take up to 5 h) can also lead to sloppy adaptation of the anatomy with scar tissue predisposing to tethering as well as CSF leakage requiring repair directly after birth. The MOMS authors recently reported a myofascial flap technique in prenatal in utero repair, because cerebrospinal fluid leakage appeared in their standard technique in nearly 50% of cases [26]. In our series, only one patient had revision due to fistula (3.2%). In the MOMS publication, 13% dehiscence at the repair site was reported [2].

Two of the eight (25.0%) status post prenatal patients managed at our clinic underwent CSF fistula repair shortly after birth. Further, 5/8 (62.5%) of these status post prenatal patients underwent hydrocephalus treatment, one of whom required 15 surgeries. Only one of those patients (12.5%) can ambulate freely. It is unclear whether those data were ever included in the respective follow-up data of the institutions that originally treated them.

## 5. Conclusions

Postnatal repair, which in this retrospective and small cohort lead to higher ambulation rate, lower secondary tethered cord surgery, lower shunt rate and lower CIC rate compared to the prenatal cohort in the MOMS trial, is still state of the art. In addition, when associated with the clearly lower rate of premature births and absence of maternal morbidity and mortality, our data suggest that postnatal surgery should still be considered the standard of care in this patient population.

## Figures and Tables

**Table 1:** Management Algorithm for MMC patients at the Altona Children’s Hospital.

Symptom Complex	Investigations
Hydrocephalus	First year of life: quarterly outpatient visits with clinical evaluation, head circumference, and transcranial ultrasound studies; Yearly outpatient checkups thereafter.
Tethered Cord	Somatosensory evoked potentials at age 3 (or when tethered cord is suspected); MRI of the complete neuroaxis at 12 and 48 months or whensymptomatic, also to rule out further NTDs, evaluate ventricle size, confirm anatomical level, and monitor syrinx.
Urological	Biannual, after 3–4 years annual outpatient checkups with ultrasound and urodynamic studies; further investigation with VCUG or scintigraphy when indicated.
Orthopedic	Yearly outpatient checkups after verticalization to assess for brace treatment, scoliosis, etc.

NTDs: open neural tube defects; VCUG: voiding cystourethrography; MRI: magnetic resonance imaging; MMC: myelomeningocele.

**Table 2:** Indications for additional surgeries.

**Hydrocephalus**	Criteria for hydrocephalus treatment were the same that were implemented in the MOMS trial [2]. In patients meeting these criteria after 12 months of age, endoscopic third ventriculostomy (ETV) was performed instead of shunt implantation.
**Secondary tethered cord**	Untethering is performed for symptomatic tethered cord, which includes loss of motor function, a reduction in somatosensory evoked potentials, progressive syringomyelia or spasticity, higher frequency of bladder infection, changes in bladder pressure, and/or progressive development of deformities of lower extremities.
**Chiari malformation**	Indication for Chiari decompression was based on cranial nerve palsy, central apnea, and progressive syringomyelia, not solely on radiological findings.
**Orthopedic surgeries**	Indications for lower limb surgery were dependent on the functional status of the children, as some are limited to ambulating patients. Spine surgeries are indicated for thoracic insufficiency syndrome or progressive scoliosis or kyphosis.
**Urologic surgeries**	Urologic surgeries were indicated for reflux, upper urinary tract deterioration, catheterization problems, and bowel management.

MOMS: Management of Myelomeningocele Study.

**Table 3:** Status post prenatal repair: a summary of patient data.

Pat. Nr.	Gestational Age at Birth (weeks)	Anatomical Level	Hydrocephalus; Shunt/ETV; (Age)	Ambulation	Urological	Other Neurosurgical Surgeries	FU (Months)
1	26	sacral	Yes; Shunt (3.5 months)	Pat. 1 year old	Antichol. Meds; no self-cath	15 CSF surgeries total	16
2	36	L2/3	Yes; ETV (16 months)	Crawls with orthosis, Pat. < 2 yrs	No meds; no self-cath	none	18
3	35	sacral	No	Freely	No meds; no self-cath	CSF fistula repair on day of birth; see Figure 4	94
4	30	L4-S1	No	With orthosis	unknown	None	67
5	31	L5	Yes; Shunt (0.5 months)	With walker + orthosis	Antichol. Meds; self-cath	None	49
6	30	T12-L1	Yes; Shunt (4 days)	Wheelchair	Antichol. Meds; no self-cath	Detethering at age 11	183
7	38	L4/5	No	With orthosis	Antichol. Meds; self-cath	None	69
8	24	L4/5	Yes; Shunt (within first year of life)		Antichol. Meds; self-cath	Detethering recently indicated	109

## Data Availability

Many data are provided in table form within the manuscript; additional data can be made available per request.
